# Changes in Serum NT-Pro BNP and Left Atrial BNP Levels after Percutaneous Transvenous Mitral Commissurotomy in Sinus Rhythm Versus Atrial Firilation

**DOI:** 10.15171/jcvtr.2014.007

**Published:** 2014-09-30

**Authors:** Leili Pourafkari, Seyedrazi Seyedhosseini, Babak Kazemi, Heydarali Esmaili, Naser Aslanabadi

**Affiliations:** ^1^Cardiovascular Research Center, Tabriz University of Medical Sciences, Tabriz, Iran; ^2^Department of Pathology, Tabriz University of Medical Sciences, Tabriz, Iran

**Keywords:** Natriuretic Pepotide, Mitral Stenosis, Percutaneous Transvenous Mitral Commissurotomy

## Abstract

*Introduction:* Natriuretic peptides are secreted from the heart in response to increased wall stress. Their levels are expected to be increased in patients with mitral stenosis (MS) due to high left atrium (LA) pressure and pulmonary artery pressure (PAP). Percutaneous transvenous mitral commissurotomy (PTMC) if successful is pursued by a rapid decrease in LA pressure and subsequent decrease in pulmonary artery pressure. The concurrent changes in natriuretic peptide levels could be affected with heart rhythm.

*Methods:* Forty five patients with severe rheumatic MS undergoing PTMC were enrolled. We evaluated the serum NT-Pro BNP levels before and 24 hours after PTMC. BNP levels were also measured from the blood samples obtained from LA before and 20 minutes after the procedure. Changes in biomarkers were assessed based on heart rhythm and success of the procedure.

*Results:* While serum NT-Pro BNP levels showed significant decrease 24 hours after the procedure (P= 0.04), BNP levels taken 20 minutes after PTMC from LA were similar to their baseline concentrations (P= 0.26). NT-Pro BNP levels decreased 51.7±182.86 pg/ml for sinus rhythm (SR) and 123.4±520 pg/ml for atrial fibrillation (AF) (P= 0.68).

*Conclusion:* Immediate changes in BNP levels did not predict the success of procedure probably due to the additional balloon inflation attempts in LA in several patients and half-life of BNP. BNP levels obtained later may be of more value considering the half-life of this marker. Heart rhythm was not found to influence the changes in biomarker levels. BNP and NT-pro BNP changes were not found to predict success of the procedure.

## Introduction

Rheumatic mitral stenosis (MS) remains a clinical problem in developing countries.^1^ Percutaneous transvenous mitral commissurotomy (PTMC) was introduced in 1984 by Inoue and since then has been performed increasingly as the treatment of choice for severe MS with favourable anatomy.^2^ Natriuretic peptides are mainly secreted from the heart and released in response to increased wall stress.^3^ BNP and NT-ProBNP are secreted both from the atria and the ventricles with the plasma half-life of 21 minutes and 60-120 minutes, respectively.^4^ NT-proBNP levels are shown to correlate positively with the severity of the stenosis in patients with MS. Following successful correction of the stenotic valve or its replacement the serum levels of this biomarker reduce significantly.^5^ Since PTMC causes rapid decreases in LA pressures and subsequent decreases in pulmonary artery pressure, it is conceivable that the natriuretic peptide concentrations change as a reflection of the hemodynamic alterations. NT-Pro BNP levels in the peripheral blood are reported to decrease one day after successful PTMC.^6,7^ Since changes in biomarker levels in LA precede serum changes, we postulated that LA sampling could provide an early indication for the success of the procedure and could offer immediate guidance for predicting favourable results. We sought to evaluate the BNP levels from left atrium (LA) before and 20 minutes after the procedure and serum NT-pro BNP levels before and 24 hours after the procedure and examine the correlation with hemodynamic outcomes of PTMC. We also compared the changes in biomarker levels in patients with sinus rhythm (SR) and atrial fibrillation (AF).

## Materials and methods

All consecutive patients with severe MS undergoing elective PTMC in Madani heart center between February 2012 and September 2013 were enrolled prospectively. All participants were approached and written informed consent was obtained by a member of the study team. Demographic and anthropomorphic data including age, gender, height and weight, body mass index (BMI) were collected and recorded. The patients were classified as in SR or AF based on their 12-lead electrocardiogram. Clinical information such as heart rhythm, history of comorbid diseases were also collected. All patients underwent transesophageal echocardiography (TEE) before PTMC. Mitral valve area (MVA) calculation was done by a combination of planimetry and doppler evaluation of tranmitral gradient and pressure half time method. Inclusion criteria were severe rheumatic MS (MVA less than 1.5 cm^2^) and the New York Heart Association (NYHA) functional class II through III. Exclusion criteria included left ventricular ejection fraction (LVEF) less than 50%, history of systemic hypertension, coexistent valvular abnormality of more than moderate severity, history of myocardial infarction or known coronary artery disease, renal failure of stage 3 or higher, severe chronic obstructive pulmonary disease, incomplete echocardiography data, mitral regurgitation of more than moderate severity, thrombus in LA or LA appendage in TEE and patients with pulmonary oedema or in NYHA functional class IV. Peripheral blood samples for measurement of NT-Pro BNP were taken from all patients before the PTMC procedure. PTMC was performed via a femoral approach with the patient under conscious sedation. Right heart catheterization was done and mitral commissurotomy was performed using an Inoue balloon catheter through transseptal approach. Five cc of blood was taken from LA right after septotomy. Successful PTMC was defined as and MVA≥ 1.5 cm^2^ or ≥ 1 cm^2^ /m^2^ without significant complications. Hemodynamic data including left atrial pressure (LAP), left ventricular end diastolic pressure and pulmonary artery pressure (PAP) were recorded before and after the procedure. Also delta-PAP (after-before) and delta-LAP (after-before) were calculated. Blood samples were taken from LA for the second measurement of BNP concentration 20 minutes after the first balloon inflation. The second measurement of plasma NT- Pro BNP was done 24 hours after the procedure. Patients underwent control transthoracic echocardiography one week later and MVA was quantified as described earlier. NT pro BNP of greater than 125 pg/ml and BNP level of greater than 100 pg/ml were considered indicator of cardiac dysfunction.

### Statistical Analysis

Data are expressed as mean ± standard deviation. Student’s *t*-test was used to determine the significance of before and after differences of paired variables. Pearson correlation coefficient analysis was used to examine the correlation between variables. P-value less than 0.05 was considered significant. All statistical analyses were done with SPSS 17 (SPSS Inc., Chicago, IL).

## Results

Fifty-four patients with severe rheumatic MS underwent PTMC during the aforementioned period. Four patients met at least one of our exclusion criteria and were excluded (One patient had LVEF less than 50%, 1 had significant aortic regurgitation, 2 had severe tricuspid regurgitation). Five patients developed more than moderate mitral regurgitation after the procedure and were subsequently excluded. Forty five patients including 12 (26.7%) males and 33 (73.3%) females met the inclusion criteria and were enrolled. The age distribution of study population was between 20 to 76 years with a mean of 44.9±14.4 years. Thirty-four (75.6%) patients were in SR and 11 (24.4%) patients had AF. Hemodynamic, MVA, BNP and NT-pro BNP before and after the procedure based on heart rhythm are shown in Table 1. There was no significant difference between patients in SR and AF in terms of MVA, LAP, PAP and biomarker levels before and after procedure. MVA quantified by echocardiography was increased from 0.94 ± 0.22 cm^2^ before PTMC to 1.68 ± 0.39 cm^2^ after the PTMC (P< 0.001). Mean increase in MVA was 0.762 cm^2^ ± 0.262 cm^2^ . LAP decreased from 28.5 ± 7.4 mmHg before procedure to 15.4 ± 6.5 mmHg after procedure (P< 0.001). PAP decreased from 45.0 ± 15.4 mmHg before PTMC to 35.8 ± 11.0 mmHg after PTMC (P< 0.001).

**Table 1:** Hemodynamic and MVA data before and after the procedure

	**Heart Rhythm**			**P**
	**NSR**	**AF**	**Total**	
MVA Before	0.92 ± 0.23	0.98 ± 0.17	0.94 ± 0.22	0.47
MVA After	1.69 ± 0.28	1.63 ± 0.62	1.68 ± 0.39	0.66
PAP Before	43.94 ± 12.77	48.36 ± 22.24	45.02 ± 15.44	0.41
PAP After	34.33 ± 8.48	40.27 ± 16.21	35.82 ± 11.02	0.12
LAP Before	28.38 ± 6.72	29.00 ± 9.86	28.53 ± 7.49	0.81
LAP After	15.24 ± 5.72	16.00 ± 9.01	15.42 ± 6.56	0.741
NT-pro BNP (After-Before)	-51.7± 182.86	-123.4±52	-67.97 ± 288.41	0.001
BNP (After-Before)	9± 72.7	175.5±86.2	49.7±295.44	0.79

NT-Pro BNP was 504.10 ± 630.81 pg/ml and 413.72 ± 423.74 pg/ml after the procedure (P= 0.04). Mean changes in NT-pro BNP (after-before) level was -67.97 ± 288.41 pg/ml. BNP levels was 124.43 ± 85.27 pg/ml before and 174.14 ± 306.30 pg/ml after the procedure (P= 0.26). Mean changes in BNP (after-before) level was 49.7±295.44 pg/ml. NT-Pro BNP levels decreased significantly after the procedure. BNP concentration failed to show a significant decrease and in most cases increased 20 minutes after the first balloon inflation.

Table 2 shows the correlation between NT-Pro BNP and BNP levels with PAP and LAP before and after PTMC. As shown NT-Pro BNP concentrations have significant relationship with PAP and LAP before and after the procedure. BNP concentrations failed to correlate to either PAP or LAP.

**Table 2:** Pearson’s correlation coefficient for BNP and NT-Pro BNP concentrations before and after the procedure.

	**PAP After**	**PAP (After-Before)**	**LAP Before**	**LAP After**	**LAP( After- Before)**
**NT- Pro BNP Before**					
Pearson	0.725	-0.110	0.346	0.402	0.020
Correlation Sig. (2-tailed)	0.000	0.482	0.022	0.007	0.896
N	43	43	44	44	44
**NT- Pro BNP After**					
Pearson	.665	-0.044	0.328	0.361	-0.003
Correlation Sig. (2-tailed)	0.000	0.780	0.030	0.016	0.985
N	43	43	44	44	44
**BNP Before**					
Pearson	0.425	0.020	0.216	0.150	-0.109
Correlation Sig. (2-tailed)	0.004	0.898	0.155	0.327	0.477
N	44	44	45	45	45
**BNP After**					
Pearson	0.192	-0.087	-0.041	-0.093	-0.052
Correlation Sig. (2-tailed)	0.212	0.574	0.787	0.543	0.736
N	44	44	45	45	45

Mean NT-Pro BNP decrease was 51.7± 182.86 for SR and 123.4±52 for AF (P= 0.678).On the other hand BNP level had an increase of 9± 72.7 pg/ml in SR and 175.5±86.2 pg/ml in AF patients (P= 0.369). Heart rhythm did not seem to influence the changes in biomarkers after the procedure.

Overall 84.4% procedures fulfilled the definition for successful procedure. Heart rhythm (P= 0.119), sex (P= 0.613) was not different among these groups. BNP level before and after the procedure and NT-pro BNP level before and after the procedure were not significantly related to the changes in MVA. Changes in NT-pro BNP was not a predictor of MVA changes (P= 0.92) or MVA after the procedure (P= 0.51). Table 3 shows the significance of different variables with regards to the success of the procedure.

**Table 3:** Evaluation of significance of different variables with regards to the success of the procedure

	**Successful**	**Not successful**	**P**
Sex (F/M)	28/10	5/2	0.61
Rhythm (NSR/AF)	27/11	7/0	0.12
NT-pro BNP>125 pg/ml before PTMC	6	33	0.59
NT-pro BNP>125 pg/ml after PTMC	5	28	0.61
BNP>100 pg/ml before PTMC	5	19	0.26
BNP>100 pg/ml after PTMC	5	21	0.36

F: Female, M: Male, NSR: Normal Sinus Rhythm, AF: Atrial Fibrillation, PTMC: Percutaneous Transvenous Mitral Commissurotomy

Regression analysis was performed to identify the variables predicting changes in NT-pro BNP (after-before). Percentage of MVA improvement, MVA after procedure, age, BMI, delta-PAP, delta-LAP and BMI were included in the analysis but none of them proved to be a predictor.

## Discussion

Rheumatic heart disease remains a major problem in the developing world.^1^ PTMC has emerged as the treatment of choice for severe rheumatic MS. With increasing expertise and proper case selection, the immediate results of the procedure are favorable and complications occur at low rates comparable to the rates reported for surgical commissurotomy.^2^

Natriuretic peptides are being increasingly used in the diagnosis and management of patients with heart failure.^8^ BNP is a polypeptide derivative of the 108 amino-acid precursor molecule named Pro-BNP which is cleaved into C-terminal (biologically active form) and N-terminal N-terminal (biologically inactive NT-proBNP).^4^ Natriuretic peptide levels are not only indicators of various cardiovascular diseases, but also are markers of their severity.^9^

Elevated plasma BNP levels are reported in patients with pure MS in SR and were found to correlate with disease severity.^10^ Changes in plasma levels of natriuretic peptides after PTMC are assessed in a few studies. In a study by Nakamura et al. on 14 patients with severe MS undergoing PTMC, plasma ANP levels decreased significantly after PTMC yet plasma BNP levels remained unchanged at both 0.5 and 24 hours after the procedure.^11^ On the other hand, Esteves et al. describe a significant decrease in plasma BNP 24 hours postvalvuloplasty on their cohort of 30 severe MS cases undergoing PTMC and report the decrease in LAP as the only predictor of a BNP levels decrease.^12^ In one study cardiac rhythm was suggested to play a possible role in BNP changes following PTMC. BNP levels declined post PTMC in patients with SR however this decrease was not reported in patients with AF.^13^ Yet such an observation was not made in the present study and heart rhythm failed to predict changes in either of the biomarker levels.

We hypothesised that changes in biomarker levels could predict success of the procedure yet our study failed to show such a correlation.

Since PTMC is performed without intra-procedural heparin in our center^14^ we could not repeat LA blood sampling more than 20 minutes after PTMC with regard to concerns of increased thrombogenicity of the prolonged procedure.

In concordance to other studies^6,7^ NT-Pro BNP levels show a significant reduction in patients after undergoing successful PTMC. The reduction correlated well with the decline observed in mean LAP and PAP.

## Conclusion

The decrease in NT-Pro BNP levels obtained 24 hours after the procedure was shown to correlate well with the observed favorable hemodynamic consequences of PTMC in this study. Immediate changes in BNP levels did not predict the success of procedure and we even observed an increase in the BNP concentration in most cases probably due to the additional balloon inflation attempts in LA in several patients and also BNP half-life. Serial BNP levels obtained later may be of more value in showing the parallel changes of this biomarker. Heart rhythm was not found to influence the changes in biomarker levels. BNP and NT-pro BNP changes were not found to predict success of the procedure in our study.

## Limitations

This study has some limitations. The main limitation was that we did not perform serial measurement of BNP 24 hours and 48 hours after the procedure. As discussed, one may not observe sudden acute changes in BNP levels parallel to the changes in LA pressure probably due to the multiple inflation attempts and BNP half life. Also we did not correlate the number of inflation attempts with the observed increase of BNP levels right after the procedure. Hemodynamic parameters including cardiac index and pulmonary vascular resistance were not evaluated in our study. Finally our study population was relatively small. Larger studies could better clarify the subject.

## Ethical issues

Research ethics committee of Tabriz University of Medical Sciences reviewed and approved of the study for its scientific merit and ethical consideration. This study was registered under the thesis number 90/3-8/19.

## Competing interests

Authors declare no conflict of interest in this study.
